# miR-34a-FOXP1 Loop in Ovarian Cancer

**DOI:** 10.1021/acsomega.3c03867

**Published:** 2023-07-18

**Authors:** Esra Dirimtekin, Maria Mortoglou, Ceren Alavanda, Asmaa Benomar Yemlahi, Esra Arslan Ates, Ilter Guney, Pinar Uysal-Onganer

**Affiliations:** † Department of Medical Genetics, School of Medicine, Marmara University, 34854 Istanbul, Turkey; ‡ Cancer Mechanisms and Biomarkers Research Group, School of Life Sciences, University of Westminster, W1W 6UW London, U.K.; § Department of Medical Genetics, Van Training and Research Hospital, University of Health Sciences, 65170 Van, Turkey; ∥ Department of Medical Genetics, Istanbul University-Cerrahpasa, Cerrahpasa Faculty of Medicine, 34098 Istanbul, Turkey

## Abstract

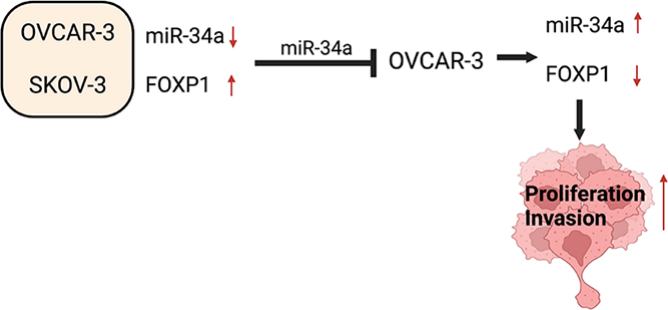

Ovarian cancer (OC) is the main cause of gynecological cancer mortality in most developed countries. microRNA (miR) expression dysregulation has been highlighted in human cancers, and miR-34a is found to be downregulated and associated with inhibition of tumor growth and invasion in several malignancies, including OC. The winged helix transcription factor forkhead box P1 (FOXP1) is reported as either an oncogene or tumor suppressor in various cancers. This study aimed to elucidate potential clinical and biological associations of miR-34a and transcription factor FOXP1 in OC. We investigated nine OC patients’ blood samples and two OC cell lines (SKOV-3 and OVCAR-3) using quantitative real-time reverse transcription polymerase chain reaction (RT-qPCR) to determine both miR-34a and FOXP1 expressions. We have found that miR-34a and FOXP1 are reversely correlated in both in vitro and in vivo. Inhibition of miR-34a transiently led to upregulation of FOXP1 mRNA expression and increased cellular invasion in vitro. Our data indicate that miR-34a could be a potential biomarker for improving the diagnostic efficiency of OC, and miR-34a overexpression may reduce OC pathogenesis by targeting FOXP1.

## Introduction

Ovarian cancer (OC) is the seventh most common malignancy worldwide, with over 295,000 new cases in 2018 and the eighth most common cause of mortality in women, with approximately 184,000 yearly deaths.^1−4^ The asymptomatic onset of the disease and the lack of robust screening methods result in the diagnosis of OC in advanced stages. In fact, according to the International Federation of Gynecology and Obstetrics (FIGO) staging system for gynecological cancers, about 70% of OC cases have progressed to the FIGO stage III and IV at the time of the diagnosis.^4,5^ Currently, cancer antigen 125 (CA125) in serum is used as a tumor biomarker to screen, detect, and manage OC.^6^ However, CA125 is not specific enough because it can be elevated in serum during the menstrual cycle, pregnancy, and pelvic inflammatory disease, which results in a considerably high number of false-positive OC diagnoses.^6,7^ Current treatment of OC consists of primary cytoreductive surgery with subsequent intravenous or peritoneal administration of platinum-based chemotherapy, which includes cisplatin and carboplatin combined with paclitaxel and docetaxel.^4,8^ Nonetheless, OC patients tend to develop chemoresistance, which leads to tumor recurrence and further contributes to the low overall survival.^9^ Epithelial OC (EOC) can be categorized based on its genetic signature and aggressiveness in type I, which is the least aggressive and tend to carry pathogenic variants (PVs) in *PTEN*, *PIK*3*CA*, *PIK*3 *BRAF*, *KRAS*, *ARID*1*A*, and *ERBB*2 genes. In contrast, type II EOC is the most aggressive and is characterized by carrying *TP*53 PVs.^10,11^

microRNAs (miRs) are noncoding, short single-stranded RNAs that are evolutionarily conserved and have been shown to modulate cellular differentiation, apoptosis, and proliferation.^12−14^ Growing evidence of transcriptomic and genomic studies has identified correlations between the aberrant expression of miR and EOC tumorigenesis.^9,15^ Specifically, the miR-34 family has gained significant attention in the past years due to its multiple potential applications in the diagnosis, prognosis, and treatment of cancer.^16,17^ The miR-34 family consists of miR-34a, encoded by a transcript located in 1p36.22, and miR-34b and miR-34c, both encoded by a common transcript in 11q23.1.^17^ The expression levels of the miR-34 family shows tissue-specificity; miR-34b/c is highly expressed in the lungs, whereas miR-34a expression is widespread across the organism.^18,19^ miR-34a has been reported in target genes transcription factors such as FOXP1, which is a member of the forkhead transcription factor family.^20,21^ The FOXP1 gene is located in chromosome 3p14.1, an area, which is associated with loss of heterozygosity in numerous tumors, indicating its potential to function as a tumor suppressor. However, in OC, FOXP1 has been classified as an oncogene.^22^ FOXP1 is involved in the development of organs such as the lung and cardiac valves, lymphocytes, and monocytes.^23,24^

High-grade serous OC has a high PV rate, and PVs in *BRCA*1/2 genes play an important role in the development of OC by creating a homologous recombination defect.^1^ The average age of OC diagnosis in patients with PVs in *BRCA*2 is 8–10 years later than in patients with PVs in *BRCA*1. Although germline *BRCA*1/2 PVs are most commonly detected in high-grade serous OC patients, it has recently been shown that limiting genetic testing to this histologic subtype will not detect all OC patients with germline *BRCA*1/2 PVs.^1^ Therefore, it is recommended that all patients with EOC should be tested for *BRCA*1/2 genes.^2^ It was reported that miR-34a expression was significantly lower in type II (high-grade serous, high-grade endometrioid, and clear cell OC) than in type I OC (low grade serous and low grade endometrioid OC).^25,26^

This study aimed to unravel the association between circulating miR-34a and FOXP1 in OC patient samples and then investigate its function by using OC cell lines.

## Results

In this study, four sets of results are presented. First, we showed miR-34a and FOXP1 expressions of OC patient samples to investigate any potential association with hereditary PVs. We then tested two epithelial OC cells to confirm potential association between miR-34a and FOXP1 expression, following this, adopting OVCAR-3 as a model cell line as it expresses a higher level of miR-34a, we transiently inhibit miR-34a expression. Finally, by using Boyden chamber invasion assay, we determined the functional role of miR-34a in OVCAR-3 cells with/out miR-34a. Overall, our results suggest that miR-34a plays a significant role in the pathophysiology of EOC.

### miR34a and FOXP1 Expressions Reversely Correlated In Vivo and In Vitro

**Figure 1:**
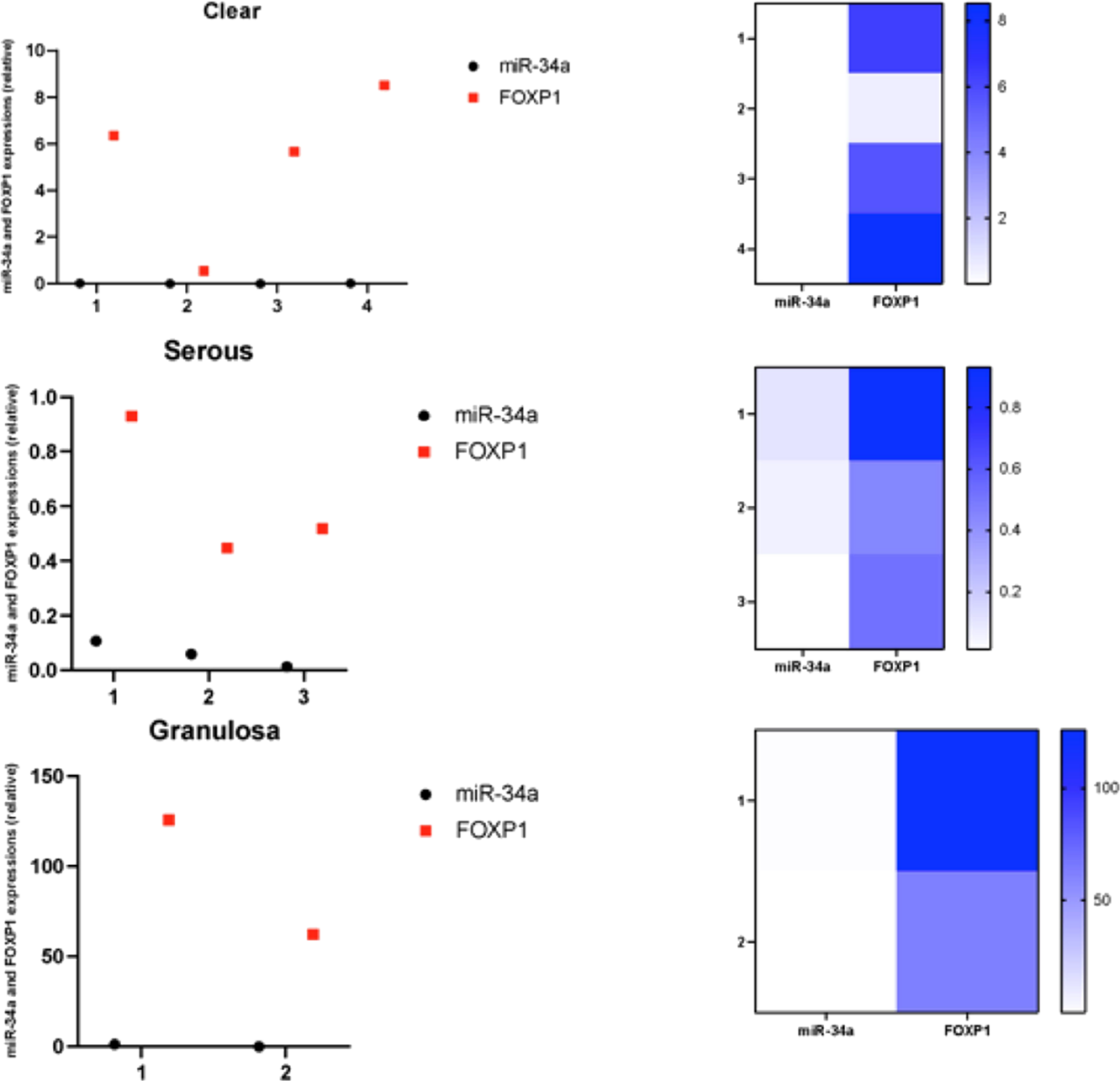
miR-34a and FOXP1 expression levels were analyzed by qRT–PCR in OC patient samples. Results were grouped in each subtypes. The black round dots represent miR-34a expressions; red squares represent FOXP1 mRNA levels. Data normalized according to RNU6 expression levels for miR-34a and RNA polymerase II (RPII) expression level for FOXP1 (*n* = 3; *p* < 0.005 for all). The heatmap was created by using GraphPad Prism (v. 9.3.1).

Nine OC patients whose consent forms were obtained were enrolled in this study. The average age of diagnosis was 50.6 ± 10.8 (mean ± SD). Histological subtypes of the OCs were epithelial (serous, *n* = 3/clear cell, *n* = 4) and sex-cord stromal (granulosa, *n* = 2), respectively. There was no patient diagnosed with germ cell OC. The FIGO stages of the patients were stage I (*n* = 1), stage II (*n* = 1), stage III (*n* = 3), and stage IV (*n* = 4), respectively. PVs were detected in three of the patients. Two of the PVs were in the *BRCA*1 gene (NM_007294.4) and one in the *RAD*51*D* (NM_002878.4) gene. We found out that a heterozygous c.5090dup (p.Leu1697Phefs*3) variant in the *BRCA*1 gene was a novel variant. Therefore, this study contributes to the PV spectrum of the *BRCA*1 gene. It is important to note that all patients with PVs had EOC. The patients’ age of diagnosis, histological subtype, FIGO stage and germline genetic analysis, and both miR-34a and FOXP1 expression results are summarized in Table 1 and Figure 1.

**Table 1:** Patient Demographics^a^

age of diagnosis	histological type	histological subtype	FIGO stage	germline genetic analysis results			miR-34a	FOXP1	FOXP1/miR34a
				variant	clinvar	ACMG			
37	epithelial	clear cell carcinoma	IV	*RAD*51*D* heterozygous c.616C > T (p.Arg206*)	P	P	0.006	5.668	944.67
40	epithelial	serous carcinoma	IV	*BRCA*1 heterozygous c.5266dup (p.Gln1756Profs*74)	P	P	0.061	0.448	7.34
41	epithelial	serous carcinoma	I	*BRCA*1 heterozygous c.5090dup (p.Leu1697Phefs*3)	not reported	LP	0.108	0.931	8.62
47	sex-cord stromal	granulosa cell tumor	IV				1.348	125.950	93.43
51	sex-cord stromal	granulosa cell tumor	IV				0.003	62.428	20809.3
54	epithelial	clear cell carcinoma	II				0.022	8.526	387545.4
55	epithelial	clear cell carcinoma	III				0.004	0.556	139
60	epithelial	clear cell carcinoma	III				0.017	6.36	374.11
71	epithelial	serous carcinoma	III				0.014	0.519	37.07

a P: pathogenic, LP: likely pathogenic.

miR-34a expression levels were found to be decreased, while FOXP1 mRNA expression was upregulated in all patients. Interestingly, the highest FOXP1 expression was detected in the granulosa cell tumor subtype of OC patients (Table 2 and Figure 1). Clear cell carcinoma patients were found to express FOXP1 mRNA more than the serous carcinoma patients. Reverse correlation of FOXP1 and miR-34a expressions were noted in all patient samples regardless of their subtypes.

**Table 2:** Patient Clinical Characteristics

age, years: median (range)	50.66 (37–71)	*n* (%)
histologic type	serous	3 (34%)
	clear	4 (44%)
	granulosa	2 (22%)
FIGO stage	I	1 (11%)
	II	1 (11%)
	III	3 (34%)
	IV	4 (44%)

**Figure 2:**
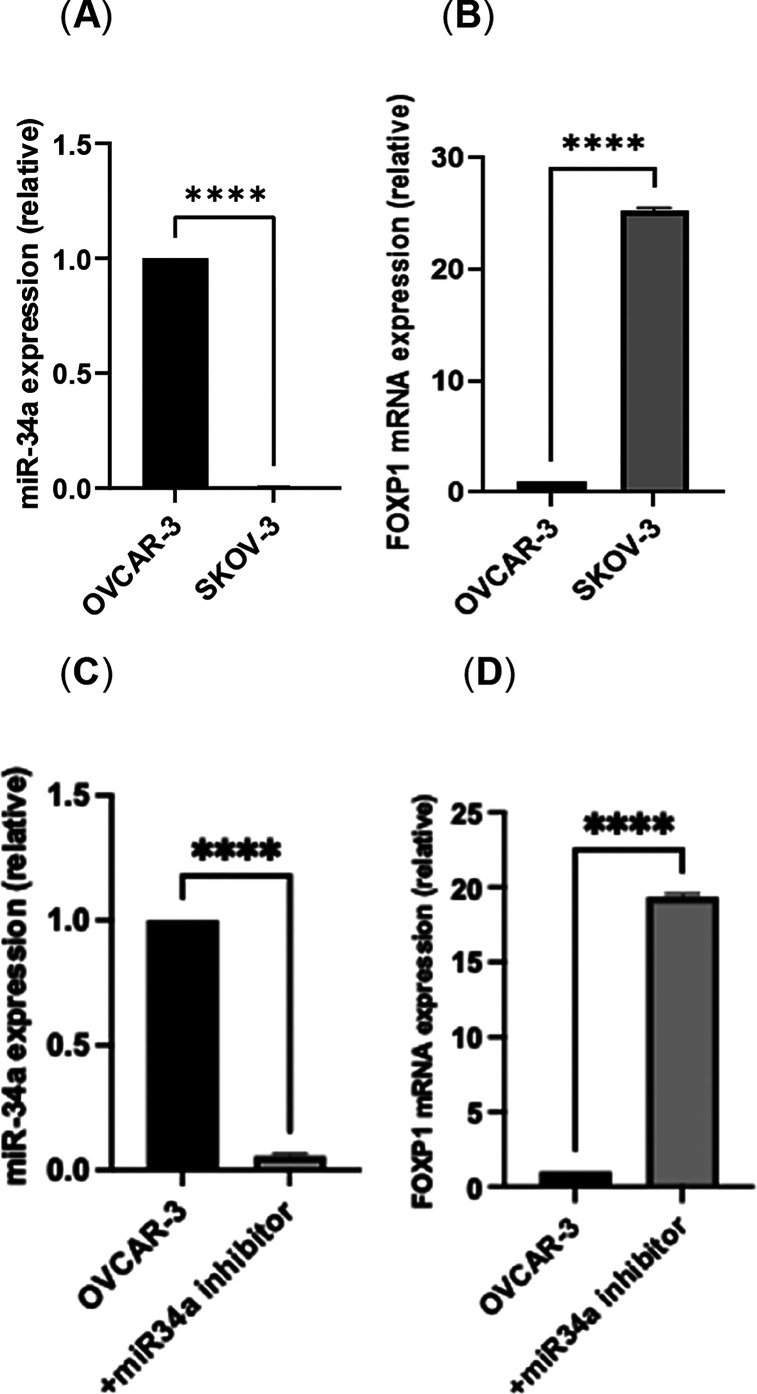
Expression levels of miR-34a and FOXP1 in SKOV-3 and OVCAR-3 cells. (A) RT-qPCR results show 143-fold lower miR-34a expression in SKOV-3 cells compared to that in OVCAR-3 cells. (B) FOXP1 mRNA level was found to be 25-fold more in SKOV-3 cells than that in OVCAR-3 cells. (C) Transiently inhibiting miR-34a in OVCAR-3 cells compared to nontransfected cells reduced miR34a expression 25-fold. (D) FOXP1 expression increased 18-fold in response to miR-34a repression in OVCAR-3 cells. The data are the mean ± SD of three technical repeats evaluated by one-way ANOVA and Bonferroni’s multiple comparison test. Exact *p*-values are indicated as *p ≤ 0.05; **p ≤ 0.01; ***p ≤ 0.001; and ****p ≤ 0.0001; error bars indicate SD.

Two epithelial OC cell lines, SKOV-3 and OVCAR-3, were tested for their miR-34a and FOXP1 expression levels. We noted that the reverse correlations between miR-34a and FOXP1 expression levels remained; however, OVCAR-3 expressed 143-fold more miR-34a than SKOV-3 cells (Figure 2A). Similarly, FOXP1 expression was found to be 25-fold less than that in OVCAR-3 cells (Figure 2B). In order to further study the potential effect of miR-34a, we transiently inhibited miR-34a expression by using OVCAR-3 as a model (Figure 2C). Inhibiting miR-34a expression led to an 18-fold increase of FOXP1 expression (Figure 2D).

**Figure 3:**
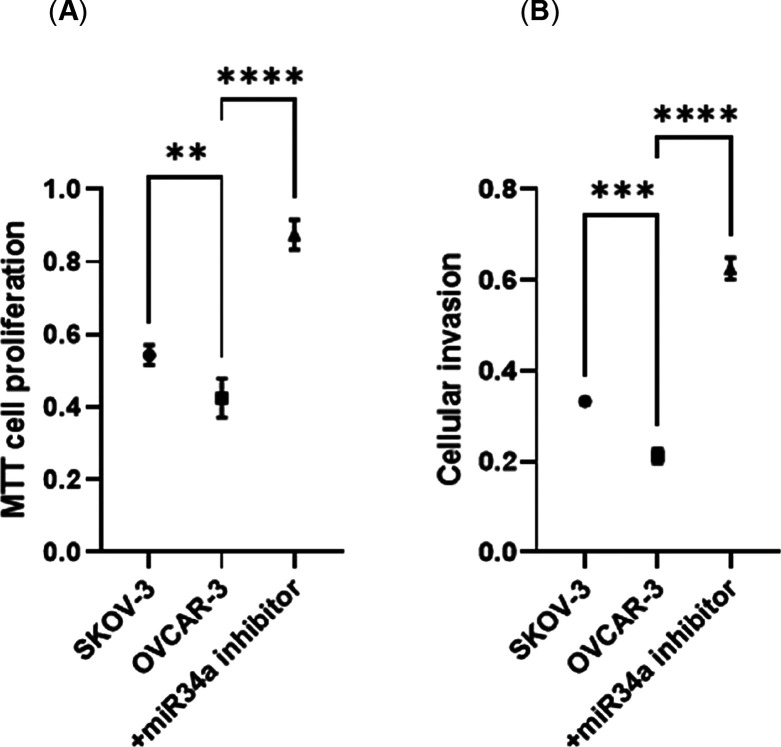
miR-34a inhibition increased cellular invasion and proliferation in OC cells. Figure (A) represents cellular proliferation of OC cells, while (B) cellular invasion of OC cells. The data are the mean ± SD of three technical repeats evaluated by one-way ANOVA and Bonferroni’s multiple comparison test. Exact *p*-values are indicated as *p ≤ 0.05; **p ≤ 0.01; ***p ≤ 0.001; and ****p ≤ 0.0001; error bars indicate SD.

We then explored the involvement of miR-34a in cellular proliferation and invasion. SKOV-3, OVCAR-3, and miR-34a-inhibited OVCAR-3 cells were tested. MTT proliferation results show that SKOV-3 cells showed 22% more proliferation than OVCAR-3 cells; however, when miR-34a was inhibited, OVCAR-3 cells proliferated 62% more when compared to SKOV-3 and 105% more when compared to nontransfected OVCAR-3 cells (Figure 3A, *n* = 3, *p* ≤ 0.01). Interestingly, when we tested the cellular invasion by using the same approach, SKOV-3 cells invaded through Matrigel 37% more than OVCAR-3 cells; however, miR-34a knockdown cells increased the cellular invasion 80% within 16 h (Figure 3B, *n* = 3, *p* ≤ 0.01).

## Discussion

The worldwide mortality of OC remains a paramount challenge due to the lack of reliable biomarkers for the early detection of OC and the late onset of unspecific symptomatology.^2−4^ Recently, miR-34a has gained attention due to its potential involvement in the pathogenesis of OC.^27^ Significant efforts have been focused on identifying target genes and key signaling and cellular pathways regulated by the tumor suppressor miR-34a.^19,28^ It was reported that low levels of miR-34a were associated with more aggressive disease and advanced-stage tumors in EOC.^29^ Moreover, it was shown that downregulation of the miR-34 family in OC is associated with more aggressive disease.^30^ FOXP1, a transcription factor, a known target of miR-34a, is identified as an oncogene that upregulated in several malignancies.^31−33^ Upregulation of FOXP1 correlated with chemoresistance in FIGO stage III serous OC and high cytoplasmic FOXP1 expression in EOC was associated with a higher tumor grade.^34^

In our study, we found that overexpression of FOXP1 reversely correlated with miR-34a expression in OC patient samples. It has been reported that *BRCA*1/2 PV carriers were diagnosed with OC at a younger age than the age at which non-PV carriers were diagnosed.^35^ As expected, patients with the PVs were diagnosed with OC at the youngest age in our cohort. Moreover, *BRCA*1/2 PV carriers were more likely to be in FIGO stage III-IV, and the pathological type of *BRCA*1/2 PVs carriers was more likely to be high-grade serous carcinoma. Hence, *BRCA*1/2 genes have been established as a critical factor in inducing EOC in patients. The fact that patients with *BRCA*1 PVs were diagnosed with EOC in our study supports this. However, we could not detect any correlation with *BRCA*1/2 PV carriers and non-PV carriers. Moreover, we did not find any significant correlations between miR-34a and/or FOXP1 expressions with PVs. Possible reasons for this may be the small number of patients, or the genes analyzed in patients do not cover all genes associated with cancer. Although the FOXP1/miR-34a ratio is higher in patients with a PV in the *RAD*51*D* gene compared to those with a *BRCA*1 PV, the main reason for this is thought to be related to the pathological type rather than the gene. Previous studies also showed that the risk of OC in women with *BRCA*1 gene PVs rises to 39–46% before the age of 70 years old, and the risk of OC in women with *BRCA*2 gene PVs increases to 10–27%.^36−48^

A similar trend was noted in OC cell lines; the SKOV-3 cell line, which is known to be more invasive presented lower expression levels of miR-34a and higher levels of FOXP1 compared to OVCAR-3 that present a less aggressive disease.^39−41^ The cooperative interplay of transcription factors and miRs has been reported to regulate the expression of cancer driver genes, tumor suppressor genes, and oncogenes to regulate the cell homeostasis.^42^ FOXP1 is upregulated in various tumors, including B-cell lymphomas, which were believed to occur as a result of chromosomal translocation.^43−45^ miR-34 and FOXP1 regulate pivotal cell processes involved in the tumorigenesis and progression of cancer, including apoptosis, cell migration metastasis, and drug resistance.^27,46^ Downregulation of miR-34a has been reported to inhibit apoptosis by increasing the expression of the B-cell lymphoma 2 (Bcl-2) and synaptotagmin 1 (SYT1) proteins in colon cancer.^47^ Moreover, reduced expression levels of miR-34a promote the growth of the tumoral cells by inhibiting apoptosis in colon cancer, while higher expression of miR-34a leads to an upregulation of apoptosis by decreasing Bcl-2 and sirtuin 1 (SIRT1), which in turn inhibited the growth of cancer cells in breast cancer.^47,48^ Moreover, miR-34a has been found to regulate the CCND1 gene, which encodes for cyclin D1, a key cell cycle regulator. Overexpression of the CCND1 gene in EOC was shown to decrease the apoptosis rate.^49,50^ miR-34a and FOXP1 have been previously reported to be involved in cancer cell proliferation; miR-34a halted B-cell development at the pro-B-cell to pre-B-cell development transition point, therefore reducing the number of mature B-cells via the inhibition of FOXP1. Complete loss of FOXP1 results in the hindering of early B-cell development, whereas the increase of FOXP1 expression by the loss of miR-34a induces an increase in the production of mature B-cells.^51^ This interaction is critical in mucosa-associated lymphoid tissue (MALT) diffuse large B-cell lymphoma (DLBCL) cells, in which it has been observed that inactivation of P53, low levels of miR-34a, elevated levels of FOXP1, and Bcl-2 overexpression are associated with unfavorable prognosis in the patients.^52^

**Figure 4:**
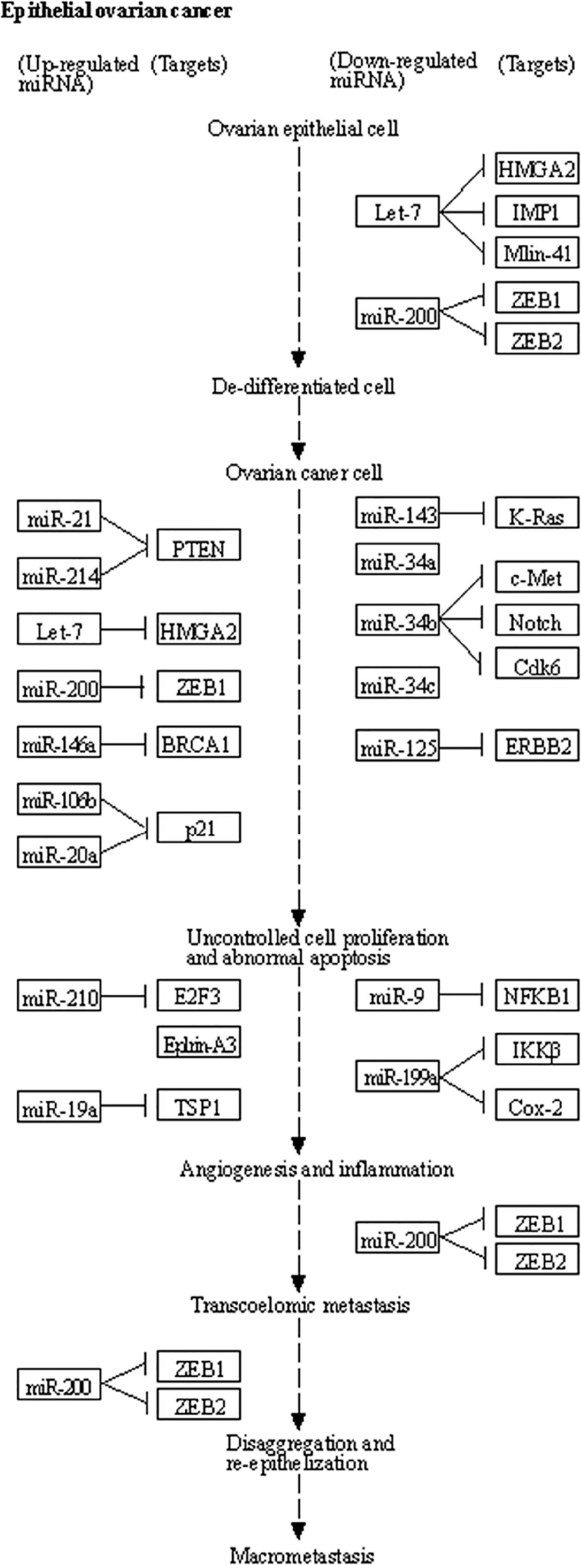
Disease pathway map for OC showing different oncogenic and tumor suppressor miRs and their associated target genes and pathways during OC development. Image taken from KEGG PATHWAY database (Kyoto, Tokyo).^9^ Accession number: map05206.

A study by Sun et al. reported that downregulation of FOXP1 expression reduced cell proliferation and invasion and increased apoptosis in glioma cells through the inhibition of the signal transducer and activator of transcription 3 (STAT3).^53^ Furthermore, a study conducted by Choi et al. indicated that FOXP1 promotes the development of OC stem cells (CSCs), increasing cell migration and drug resistance.^22^ In our study, we have found that there is a strong correlation between miR-34a inhibition and FOXP1 mRNA expression levels in OVCAR-3 cells. Epithelial-mesenchymal transition (EMT) and mesenchymal-epithelial transition (MET) are primary mechanisms for EOC progression, cellular invasion, and metastasis. These pathways are mainly regulated by direct target genes of miR-34a, which include *SNAIL*1, *TWIST*, and *ZEB*1 genes.^54^Figure 4 provides a detailed summary of the major miRs and their related target genes involved in different stages of OC development as obtained from the KEGG PATHWAY database. However, additional studies are essential to further understand the synergistic mechanisms of miR-34a and FOXP1 in OC tumorigenesis. A further study by Yao et al. reported that miR-34a upregulation decreased the cellular viability of SKOV-3 and OV-90 cells, compared to resveratrol.^55^ In our study, we identified that inhibition of miR-34a in OVCAR-3 cells resulted in a higher tendency of these cells to invade compared to OVCAR-3 and SKOV-3 cells. In addition, we have shown promising insights for the potential use of miR-34a as a diagnostic biomarker between OC subtypes. Recent studies have demonstrated that tumor-derived miRs, which are present in a stable form in human serum and plasma, can be used for investigation of these blood-based biomarkers.^56,57^ In OC, it has been shown previously that eight miRs (miR-21, miR-92, miR-93, miR-126, miR-29a, miR-155, miR-127, and miR-99b) can distinguish between normal and patient serum.^58^ Therefore, previous studies in miR profiling have suggested that patient sera could potentially be used as OC diagnostic biomarkers.^9^ However, miR diagnostic profiling in OC is a new investigation area, which needs a uniform technical platform and standard protocols between researchers to enable clinical practices.

## Conclusions

This study outlined the potential cross-talks between miR-34a and FOXP1 in the tumorigenesis of EOC. Low expression of miR-34a and high expression of FOXP1 correlated with a more aggressive OC cell line phenotype. Moreover, the inhibition of miR-34a led to the upregulation of FOXP1, confirming FOXP1 to be a direct target of miR-34a. Finally, underexpression of miR-34a was found to enhance the viability of EOC cells. Further research investigating the miR-34a-FOXP1 network is imperative to determine their specific role in the cellular processes that drive the tumorigenesis before considering their use as diagnostic and prognostic biomarkers and targets in therapeutics in EOC.

## Methodology

### Clinical Samples and DNA Extraction

Patients diagnosed with OC were included in the study. DNA was extracted from peripheral blood samples of the patients with the QIAamp DNA Mini Kit (Qiagen, MD, USA) for germline genetic testing. Both single nucleotide variants and copy number variants of 25 genes related to OC (*BRCA*1, *BRCA*2, *BRIP*1, *CDH*1, *CHEK*2, *NBN*, *PALB*2, *PIK*3*CA*, *FAM*175*A*, *MRE*11*A*, *RAD*50, *RAD*51*C*, *RAD*51*D*, *TP*53, *XRCC*2, *ATM*, *BARD*1, *MLH*1, *MSH*2, *MSH*6, *MUTYH*, *PMS*2, *APC*, *PTEN*, and *STK*11) were sequenced by the Illumina NextSeq platform (Illumina Inc., San Diego, CA, USA). The data were analyzed using the SOPHiA DDM analysis platform (SOPHiA Genetic Inc. Boston, MA 02116, USA). Variants were evaluated according to the American College of Medical Genetics and Genomics (ACMG) criteria.^59^

### Ethics

Ethical permission for the conduction of the study was obtained from the institutional ethics committee (Marmara University, Medical School, Ethics Committee 455/030323). Patients were staged according to the FIGO staging system for ovarian tumors (Table 2).

### Cell Culture

SKOV-3 (ATCC HTB-77, 2022) and OVCAR-3 (ATCC HTB-75, 2022) OC cell lines were obtained from ATCC, cultured in Dulbecco’s Modified Eagle’s Medium (DMEM), supplemented with 10% fetal bovine serum (FSB) (HyClone, Fisher Scientific, Hemel Hempstead, UK), 100 U/mL penicillin, and 100 μg/mL of streptomycin (Invitrogen, Waltham, MA, USA). Both cell lines were maintained in a humidified incubator with 5% CO_2_ at 37 °C (Heracell 150i, Thermo Fisher Scientific, Waltham, MA, USA). Anti-miR-34a (60 nM; Integrated DNA Technologies, Coralville, IA, USA) transfection was performed on the OVCAR-3 cell line by Lipofectamine 2000 (Thermo Fisher Scientific, Waltham, MA, USA) according to the manufacturer’s protocol.

### RNA Extraction and RT-qPCR

RNA was extracted from cells using Trizol (Sigma-Aldrich, Haverhill, UK), and RNA concentration and purity were measured using a NanoDrop spectrophotometer (Thermo Fisher Scientific, Hemel Hempstead, UK) at 260 and 280 nm absorbance. Reverse transcription of RNA to cDNA was carried out using a miRCURY LNA RT Kit (Qiagen, Manchester, UK) according to the manufacturer’s instructions. miRCURY LNA miRNA SYBR Green (Qiagen, Manchester, UK) was used in conjunction with MystiCq microRNA qPCR primers for miR-34a (Sigma-Aldrich, Haverhill, UK). The expression levels of miR-34a were normalized to that of U6 using the 2^ΔΔCT^ method.^60^ The sequences for U6 primers were forward 5′- GCTTCGGCAGCACATATACTAAAAT-3′ and reverse 5′- CGCTTCACGAATTTGCGTGTCAT-3′. The RT-qPCR conditions for miR-34a were as follows: heat activation at 95 °C for 2 min, followed by 40 cycles of denaturation at 95 °C for 10 s and combined annealing/extension at 56 °C for 60 s.

cDNAs for the analysis of FOXP1 expression were generated using qScript cDNA SuperMix (Quantabio, UK) with incubations at 42 °C for 30 min and 85 °C for 5 min. The FOXP1 gene expression was analyzed by using PrecisionPLUS qPCR Master Mix (PrimerDesign, UK) for RT-qPCR synthesis with the following thermocycling conditions for 40 cycles: 95 °C for 2 min, 95 °C for 10 s, and 60 °C for 60 s. The relative expression of FOXP1 was calculated with RPII. The sequences for FOXP1 primers were forward 5′- CAGTGGTAACCCTTCCCT T-3′ and reverse 5′- CGTTCAGCTCTTCCCGTA-3′.

RPII primers were forward 5′- GCACCACGTCCAATGACAT-3′ and reverse 5′- GTGCGGCTGCTTCCATAA-3′.

### Assays for Cellular Invasion and Proliferation

The anti-miR-34a-transfected OVCAR-3 cells along with SKOV-3 and wt OVCAR-3 cells were seeded at a density of 1 × 10^4^ cells per well in 96-well plates and the MTT proliferation assay was performed following further 48 h incubation. Cell invasion assays were performed as described before.^61^ Following 48 h incubation of anti-miR-34a transfection, 5 × 10^5^ cells per cell line were plated on Matrigel-coated Transwell filters (BD Biosciences, Nottingham, UK) in a chemotactic gradient of 1:10% FBS. After 16 h, the total number of invaded cells was determined by MTT assay, and this was confirmed by crystal violet assay. In parallel, the same number of were cells plated and incubated for 16 h to determine the effect of cell proliferation by MTT assay.

### Data Analysis

All data were analyzed as means ± standard errors. Statistical significance was determined using a Student’s *t*-test or ANOVA with a Newman–Keuls post-hoc analysis, as appropriate. Results were considered significant for *p* < 0.05. One-way ANOVA Bonferroni’s multiple comparison test was performed using GraphPad Prism version 7.00 for Windows (GraphPad Software, La Jolla, CA, USA) www.graphpad.com.
